# Vaccination with BCGΔBCG1419c protects against pulmonary and extrapulmonary TB and is safer than BCG

**DOI:** 10.1038/s41598-021-91993-8

**Published:** 2021-06-14

**Authors:** Michel de Jesús Aceves-Sánchez, Mario Alberto Flores-Valdez, César Pedroza-Roldán, Elizabeth Creissen, Linda Izzo, Fabiola Silva-Angulo, Clinton Dawson, Angelo Izzo, Helle Bielefeldt-Ohmann, Cristian Alfredo Segura-Cerda, Wendy López-Romero, Jorge Bravo-Madrigal, Jorge Alberto Barrios-Payán, Miguel Ángel de la Cruz, Miguel Ares, María Guadalupe Jorge-Espinoza

**Affiliations:** 1 Biotecnología Médica y Farmacéutica, Centro de Investigación y Asistencia en Tecnología y diseño del Estado de Jalisco, Av. Normalistas 800, Col. Colinas de la Normal, 44270 Guadalajara, Jalisco Mexico; 2 Departamento de Medicina Veterinaria, Centro Universitario de Ciencias Biológicas y Agropecuarias, Universidad de Guadalajara, Zapopan, Mexico; 3 Department of Microbiology, Immunology and Pathology, Colorado State University, Fort Collins, CO USA; 4 School of Chemistry and Molecular Biosciences, University of Queensland St. Lucia Campus, St Lucia, QLD 4072 Australia; 5 Laboratorio de Patología Experimental, Instituto Nacional de Ciencias Médicas y Nutrición Salvador Zubirán, Vasco de Quiroga 15, Belisario Domínguez sección 16, Tlalpan, Ciudad de México, Mexico; 6 Unidad de Investigación Médica en Enfermedades Infecciosas y Parasitarias, Centro Médico Nacional (CMN) Siglo XXI, Instituto Mexicano de Seguro Social (IMSS), Ciudad de México, Mexico

**Keywords:** Live attenuated vaccines, Bacterial pathogenesis

## Abstract

A single intradermal vaccination with an antibiotic-less version of BCGΔBCG1419c given to guinea pigs conferred a significant improvement in outcome following a low dose aerosol exposure to *M. tuberculosis* compared to that provided by a single dose of BCG Pasteur. BCGΔBCG1419c was more attenuated than BCG in murine macrophages, athymic, BALB/c, and C57BL/6 mice. In guinea pigs, BCGΔBCG1419c was at least as attenuated as BCG and induced similar dermal reactivity to that of BCG. Vaccination of guinea pigs with BCGΔBCG1419c resulted in increased anti-PPD IgG compared with those receiving BCG. Guinea pigs vaccinated with BCGΔBCG1419c showed a significant reduction of *M. tuberculosis* replication in lungs and spleens compared with BCG, as well as a significant reduction of pulmonary and extrapulmonary tuberculosis (TB) pathology measured using pathology scores recorded at necropsy. Evaluation of cytokines produced in lungs of infected guinea pigs showed that BCGΔBCG1419c significantly reduced TNF-α and IL-17 compared with BCG-vaccinated animals, with no changes in IL-10. This work demonstrates a significantly improved protection against pulmonary and extrapulmonary TB provided by BCGΔBCG1419c in susceptible guinea pigs together with an increased safety compared with BCG in several models. These results support the continued development of BCGΔBCG1419c as an effective vaccine for TB.

## Introduction

Tuberculosis (TB) continues to be a significant global public health problem. In 2019, approximately 10 million cases of active TB and 1.4 million deaths occurred worldwide^1^. It has been estimated that 1–2 billion people are latently infected with *Mycobacterium tuberculosis* (*M. tuberculosis*), the ethiological agent of TB^2^. Approximately 5–10% of individuals who get infected with *M. tuberculosis *(via inhalation of aerosol droplets),  develop symptomatic, primary pulmonary TB, with 90–95% of people infected showing no clinical signs of disease, and develop asymptomatic, latent infection (LTBI). Transmission of *M. tuberculosis* occurs during late stages of TB after extensive lung tissue damage with varying degrees of cavitation. During this process, *M. tuberculosis* gains access to the airways and is expelled, so it can start a new transmission cycle^3–5^. BCG protects children against meningeal and miliary tuberculosis when administered as a prevention of disease vaccine to non-infected children, and it has recently been shown that the revaccination with BCG to humans with LTBI (a prevention of disease vaccine in infected humans) can reduce the rate of Quantiferon^®^ conversion^6^, which might result from the capacity to either reduce reactivation from LTBI or to reduce new infections. To improve control of TB, over 20 novel vaccine candidates have been characterized in recent years, with two of them, live, whole cell attenuated MTBVAC and VPM1002, already in clinical trials. Our group developed the vaccine candidate BCGΔBCG1419c, based on the hypothesis that biofilms mimic unexplored aspects of TB pathology^7^, therefore increasing biofilm production by BCG by deleting the *BCG1419c* gene^8^, which encodes for a phosphodiesterase of the second messenger c-di-GMP. To date, using a hygromycin-resistant (Hyg^R^) version of this vaccine candidate^8^, we have shown its improved efficacy compared with parental BCG in reducing chronic TB and reactivation of disease in infected hosts, in BALB/c and B6D2F1 mice, respectively^9^. We also found that in C57BL/6 mice, BCGΔBCG1419c Hyg^R^ controlled *M. tuberculosis* replication in lungs as efficiently as parental BCG, with an improvement in reducing lung pathology and proinflammatory cytokines in lungs^10^. Based on these findings, we developed a second-generation version of BCGΔBCG1419c, devoid of antibiotic markers. Changes in the production of cellular and secreted proteins by this novel BCGΔBCG1419c compared with parental BCG were recently reported^11^, and found to match changes in antigenic proteins reported for the Hyg^R^ first-generation mutant^12^. Using the antibiotic-less version of BCGΔBCG1419c, here we compared its in vitro growth, its replication in murine macrophages, its safety in immunocompetent hosts (BALB/c and C57BL/6 mice, and Hartley guinea pigs) and immunocompromised hosts (athymic nu/nu mice), to that of parental BCG Pasteur. Considering that both C57BL/6 and BALB/c mice are resistant to *M. tuberculosis* infection and do not produce caseous granulomas in the lungs, unlike typical lesions found in human TB disease^13,14^, here we employed the guinea pig model for evaluation of vaccine efficacy, which are highly susceptible to *M. tuberculosis* infection and whose reliability and reproducibility in obtaining efficacy data was independently confirmed in three different laboratories^15^. Comparison of efficacy in guinea pigs showed that the antibiotic-less version of BCGΔBCG1419c provided improved control of both pulmonary and extrapulmonary TB compared with its parental BCG.

## Results

### In vitro characterization of the novel BCGΔBCG1419c

**Figure 1:**
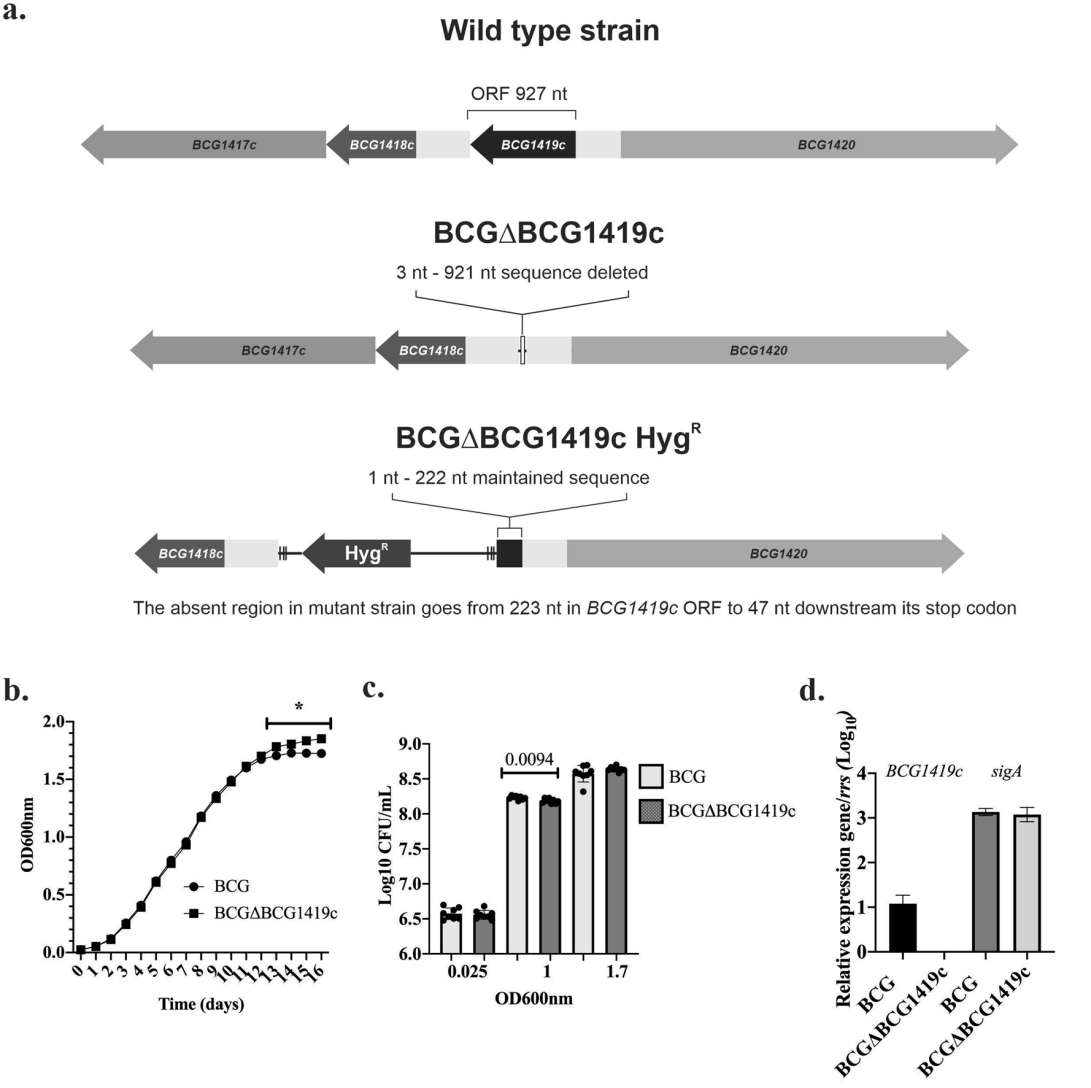
Characterization of the novel, antibiotic-less version of the TB vaccine candidate BCGΔBCG1419c. (**a**) Schematic representation of the *BCG1419c* gene and flanking regions present in wild type BCG, the antibiotic-less, and the hygromycin resistant (Hyg^R^) versions of BCGΔBCG1419c. (**b**) Growth curve of BCG and BCGΔBCG1419c. An asterisk (*) denotes a statistically significant difference (p < 0.05) between OD600nm readings of BCG and BCGΔBCG1419c from day 11 to day 16, as evaluated with multiple t tests corrected for multiple comparison with Holm-Sidak method at α = 0.05. Data (n = 4) is shown as mean with standard deviation (SD). (**c**) Bacterial replication determined as colony-forming units (CFU)/mL at 3 different OD_600_ (0.025, 1.0, 1.7). Mean Log_10_ CFU/mL with individual data (n = 8 per BCG strain) with SD are shown, and were compared with an unpaired, two-tailed Student’s t test, with Welch’s correction when standard deviation of the groups was different. (**d**) Comparison of *BCG1419c* and *sigA* gene expression in wild type BCG and BCG∆BCG1419c. Mean Log_10_ gene expression relative to *rrs* with SD is shown for each strain (n = 6 per BCG strain), a Mann–Whitney test was used for comparison.

Considering that attenuation without the presence of antibiotic-resistance markers is required to fulfill the Geneva consensus criteria^16^, we constructed a novel, antibiotic-less version of the BCGΔBCG1419c vaccine candidate (Fig. 1a**,** indicating differences compared with wild type and the Hyg^R^ version), and proceeded to evaluate its apparent growth (OD600nm), replication (CFU enumeration), and expression of *BCG1419c* and *sigA *in vitro compared to its parental BCG. Here, we observed a significant difference in apparent growth of BCGΔBCG1419c in stationary phase cultures (days 13–16, Fig. 1b). As for replication, BCG showed higher CFU than BCGΔBCG1419c at OD600nm 1 (p = 0.0094, Fig. 1c) but this difference was not observed when cultures reached OD600nm 1.7 (Fig. 1c, onset of stationary phase, Fig. 1b). As for gene expression, we confirmed the lack of *BCG1419c* transcripts in BCGΔBCG1419c whereas this mutant had similar expression of *sigA* to that observed for BCG (Fig. 1d).

### Vaccination with BCGΔBCG1419c is safer than parental BCG Pasteur in mice

**Figure 2:**
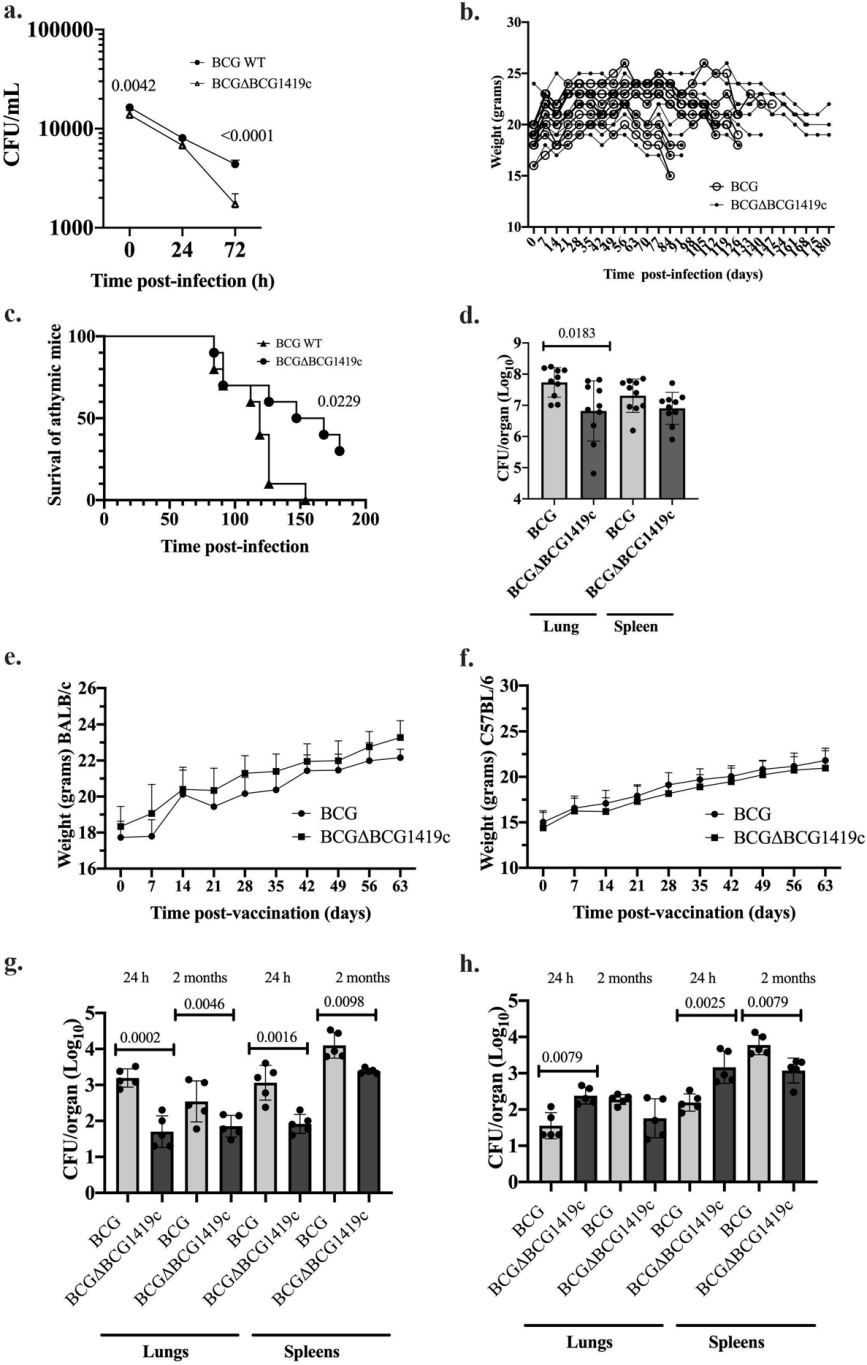
The BCGΔBCG1419c vaccine candidate is more attenuated than parental BCG. (**a**) Kinetics of murine macrophage infection assay in RAW 264.7 using BCG or BCG∆BCG1419c. Data (n = 6 per BCG strain/time point) is shown as mean with SD. A One-Way ANOVA followed by Tukey’s multiple comparison test or a Brown-Forsythe and Welch ANOVA followed by Dunnett’s multiple comparison test were used depending on SDs being or not different among groups compared, and significantly different p values indicated. (**b**) Body weight registry of individual athymic mice (n = 10/group) intravenously infected with 10^6^ CFU of BCG or BCGΔBCG1419c, used to inform humane endpoint (≥ 20% of weight loss). (**c**) Survival of athymic mice at 180 days post intravenous infection, with p value resulting from the Log-rank (Mantel-Cox) test shown. (**d**) Bacterial replication in lungs and spleen of athymic mice euthanized when they lost ≥ 20% of its maximum weight. Mean Log_10_ CFU/organ with individual data (n = 10 per BCG strain) with SD are shown, and were compared with an unpaired, two-tailed Student’s t test, with Welch’s correction when standard deviation of the groups was different, with significantly different p values indicated. (**e**) Body weight registry of individual BALB/c mice (n = 10/group) subcutaneously vaccinated with 10^7^ CFU BCG or BCGΔBCG1419c over 2 months (63 days). (**f**) Body weight registry of individual C57BL/6 mice (n = 10/group) subcutaneously vaccinated with 10^7^ CFU BCG or BCGΔBCG1419c over 2 months (63 days). (**g**) Bacterial replication in lungs and spleen of BALB/c mice (n = 5 per time point) at 24 h and 2 months (63 days) post-vaccination. Mean Log_10_ CFU/organ with individual data (n = 5 per BCG strain) with SD are shown, and were compared with an unpaired, two-tailed Student’s t test, with Welch´s correction when standard deviation of the groups was different, with significantly different p values indicated. (**h**) Bacterial replication in lungs and spleen of C57BL/6 mice (n = 5 per time point) at 24 h and 2 months (63 days) post-vaccination. Mean Log_10_ CFU/organ with individual data (n = 5 per BCG strain) with SD are shown, and were compared with an unpaired, two-tailed Student’s t test, with Welch’s correction when standard deviation of the groups was different, with significantly different p values indicated.

To evaluate the attenuation of the novel version of BCGΔBCG1419c, we first evaluated its replication compared with that of parental BCG in the murine macrophage cell line RAW 264.7. There, we observed that since initial interaction (6 h incubation, considered time zero post-infection as already described for the Hyg^R^ version of BCGΔBCG1419c^12^), antibiotic-less BCGΔBCG1419c had a 16% lower mean CFU than BCG (p = 0.0042, unpaired Student t test), difference that was more pronounced at 72 h post-infection (60% lower mean CFU, p < 0.0001, unpaired Student t test, Fig. 2a).

We next applied 10^6^ CFU via i.v. of BCGΔBCG1419c or BCG to athymic mice, followed their weight gain over time, and determined their median survival time (MST). Individual weight was recorded on a weekly basis and the loss of ≥ 20% of its maximum weight was used as humane endpoint for euthanasia (Fig. 2b). We observed that mice that received BCGΔBCG1419c had an MST of 157.5 days, compared with 119 days for those where BCG was administered [p = 0.0229, Log-rank (Mantel-Cox) test, Fig. 2c], indicating that the antibiotic-less version of BCGΔBCG1419c was more attenuated than its parental BCG in immunocompromised mice. To further assess attenutation, we determined CFU in whole lungs and spleens of these mice, where the mean loads of BCGΔBCG1419c in lungs were significantly lower than those of BCG (Log_10_ 6.8 versus Log_10_ 7.7, respectively, p = 0.0183, unpaired t test with Welch's correction, Fig. 2d), while their mean burden in spleens were not significantly different (Log_10_ 6.9 versus Log_10_ 7.3, respectively, p = 0.1018, unpaired t test, Fig. 2d).

We also evaluated weight gain of BALB/c (Fig. 2e) and C57BL/6 mice (Fig. 2f) over 2 months, as well as replication of BCG and BCGΔBCG1419c in lungs and spleens of these mice. For this, animals received 2.5 × 10^7^ CFU, s.c., and bacterial burdens were determined at 24 h and 2 months post-vaccination. Regarding weight gain, we found no difference between animals that received either BCG or BCGΔBCG1419c (Fig. 2e,f). Regarding bacterial replication, in BALB/c mice, at 24 h, BCG had 1.5-log_10_ higher CFU than BCGΔBCG1419c in lungs (p = 0.0002, unpaired, two-tailed t test), a difference that was reduced to 0.7-log10 at 2 months yet it still was significantly different (p = 0.0046, unpaired, two-tailed t test; Fig. 2g). In spleens, BCGΔBCG1419c loads were lower than those observed for BCG, both at 24 h (1.2-log_10_, p = 0.0016, unpaired, two-tailed t test) and 2 months (0.7-log_10_, p = 0.0098, unpaired t test with Welch's correction, Fig. 2g).

On the other hand, in C57BL/6 mice, BCGΔBCG1419c colonized better than BCG both lungs and spleen at 24 h, with 1-log_10_ more CFU in both organs (p = 0.0079, Mann–Whitney, two-tailed test, and p = 0.0025 by unpaired, two-tailed t test, Fig. 2h). At 2 months post-vaccination, BCGΔBCG1419c replicated less than BCG in lungs (0.5-log_10_) although this difference did not reach significance (p = 0.0977, unpaired t test with Welch's correction). In spleens, BCGΔBCG1419c maintained a significantly lower replication than BCG (0.5-log_10_, p = 0.0079, Mann–Whitney test, Fig. 2h). Taken together, these data show that the novel version of BCGΔBCG1419c was more attenuated than its parental BCG in murine RAW 264.7 macrophages as well as in athymic and immunocompetent mice.

### Vaccination with BCGΔBCG1419c is as safe as parental BCG Pasteur and induces higher PPD-reactive antibodies than BCG in guinea pigs

**Figure 3:**
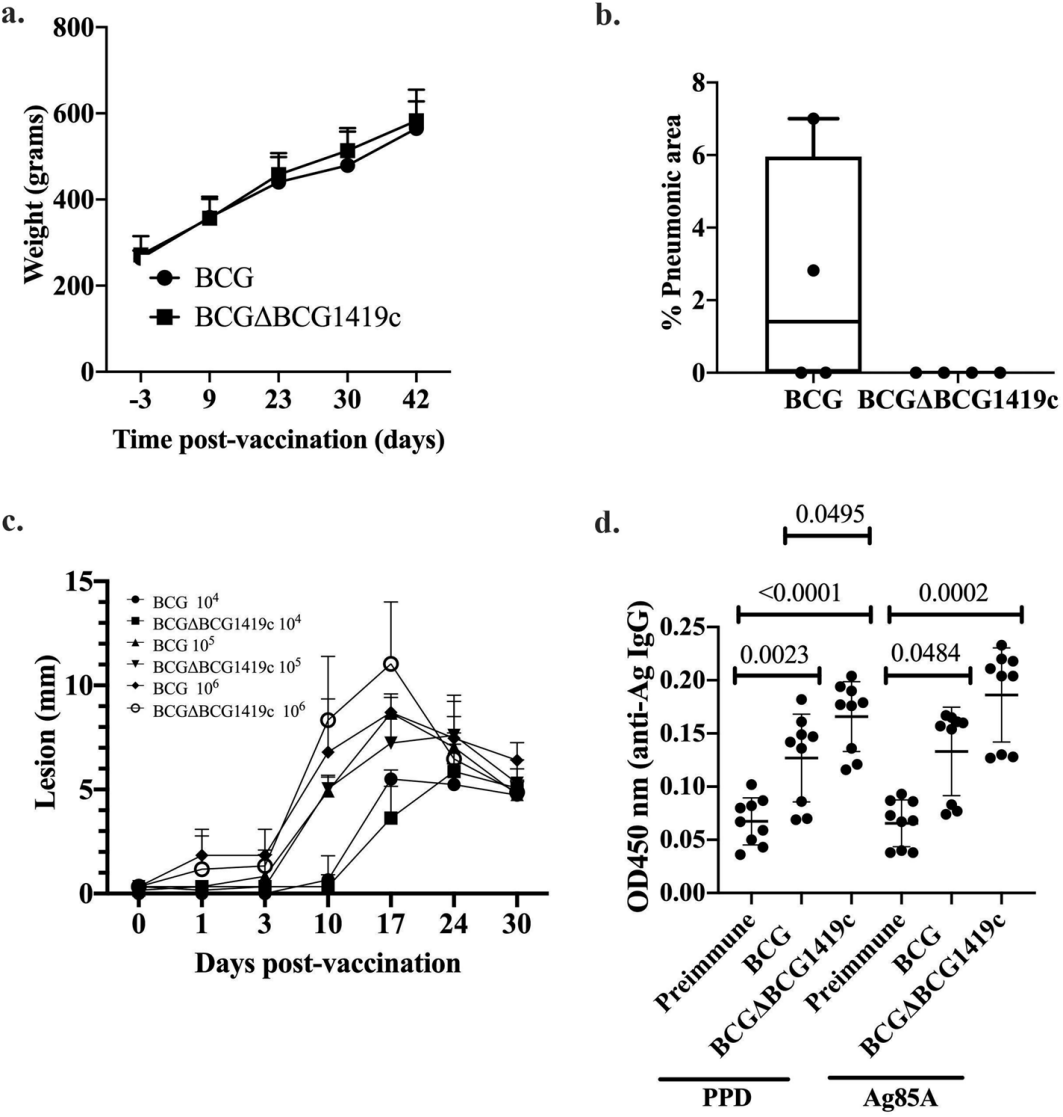
The BCGΔBCG1419c vaccine candidate is as safe as parental BCG and induces more anti-PPD IgG. (**a**) Body weight registry of guinea pigs (n = 6/group) after intramuscular (i.m.) vaccination with 10^7^ CFU of BCG or BCGΔBCG1419c over 42 days. Data is presented as means with SD and were evaluated with multiple t tests corrected for multiple comparison with Holm–Sidak method at α = 0.05. (**b**) Histopathological assessment of pneumonic areas found at 42 days post-vaccination (i.m.) in guinea pigs (n = 4/group) that received 10^7^ CFU of BCG or BCGΔBCG1419c. Data is presented as a box and whisker plot showing all points. (**c**) Excessive dermal reactivity in guinea pigs (n = 6/group). Each guinea pig was intradermally injected with three different doses of BCG or BCGΔBCG1419c. Lesions formed at the sites of injection were observed over a 28-day period. In each animal, the papule sizes resulting from vaccination with either BCG strain were compared with a multiple t test corrected for multiple comparison with Holm–Sidak method at α = 0.05. Data are presented as mean with SD. (**d**) Anti-IgG reactivity to bovine PPD and recombinant Ag85A from guinea pigs (n = 9/group) pre-vaccination (preimmune) and 10 weeks post-vaccination with 10^3^ CFU of BCG or BCGΔBCG1419c. Data are presented as mean with SD with individual OD450nm values shown. A One-Way ANOVA followed by Tukey’s multiple comparison test or a Kruskal–Wallis followed by Dunn’s multiple comparison test were used depending on distribution of data, and significantly different p values are indicated.

To support further development of the novel version of BCGΔBCG1419c, we performed tests to evaluate its safety according to the WHO Recommendations to Assure the Quality, Safety and Efficacy of BCG vaccines^17^, which here consisted in evaluating the absence of virulent mycobacteria and comparing dermal reactivity in guinea pigs vaccinated with BCGΔBCG1419c or BCG. The first safety test is used to ensure that there are no signs of TB symptoms, that animals gain weight and that upon autopsy, they do not reveal any sign of TB infection. In our observation period of time (42 days), all animals gained weight regardless of the BCG applied at a 1.5 × 10^7^ CFU intramuscular dose (Fig. 3a), being well and healthy without any abnormal behavior throughout the experiment. Post-mortem examination of all guinea pigs at day 42 showed between 0 and 8% pneumonic area for animals vaccinated with BCG, while there were no signs of TB-like lesions in the lungs of those that received BCGΔBCG1419c (Fig. 3b).

Regarding the comparison of dermal reactivity, this safety test is used to determine any potential excessive reaction given that BCG is applied intradermally in humans, where we observed no differences in lesions produced by BCG or BCGΔBCG1419c at any of the three doses employed (Fig. 3c). Together, these results demonstrate that BCGΔBCG1419c is at least as safe as parental BCG in guinea pigs.

As the role of B lymphocytes and antibodies in TB has recently been revisited^18^, we obtained sera from guinea pigs at 10 weeks post-vaccination with 10^3^ CFU of either BCG or BCGΔBCG1419c, and evaluated their capacity to recognize PPD or recombinant Ag85A in ELISA assays. There, we found that anti-PPD IgG OD450 nm readings were higher in BCGΔBCG1419c-vaccinated animals than in those vaccinated with BCG (Fig. 3d, p = 0.0495, One-Way ANOVA with Tukey’s multiple comparison test). For recombinant Ag85A, both BCG and BCGΔBCG1419c induced more IgG than unvaccinated (pre-immune) guinea pigs (Fig. 3d, p = 0.0484 and p = 0.0002, Kruskal–Wallis with Dunn’s multiple comparison test) but with no difference between BCG strains tested.

### BCGΔBCG1419c is more effective than BCG in reducing pulmonary TB pathology

**Figure 4:**
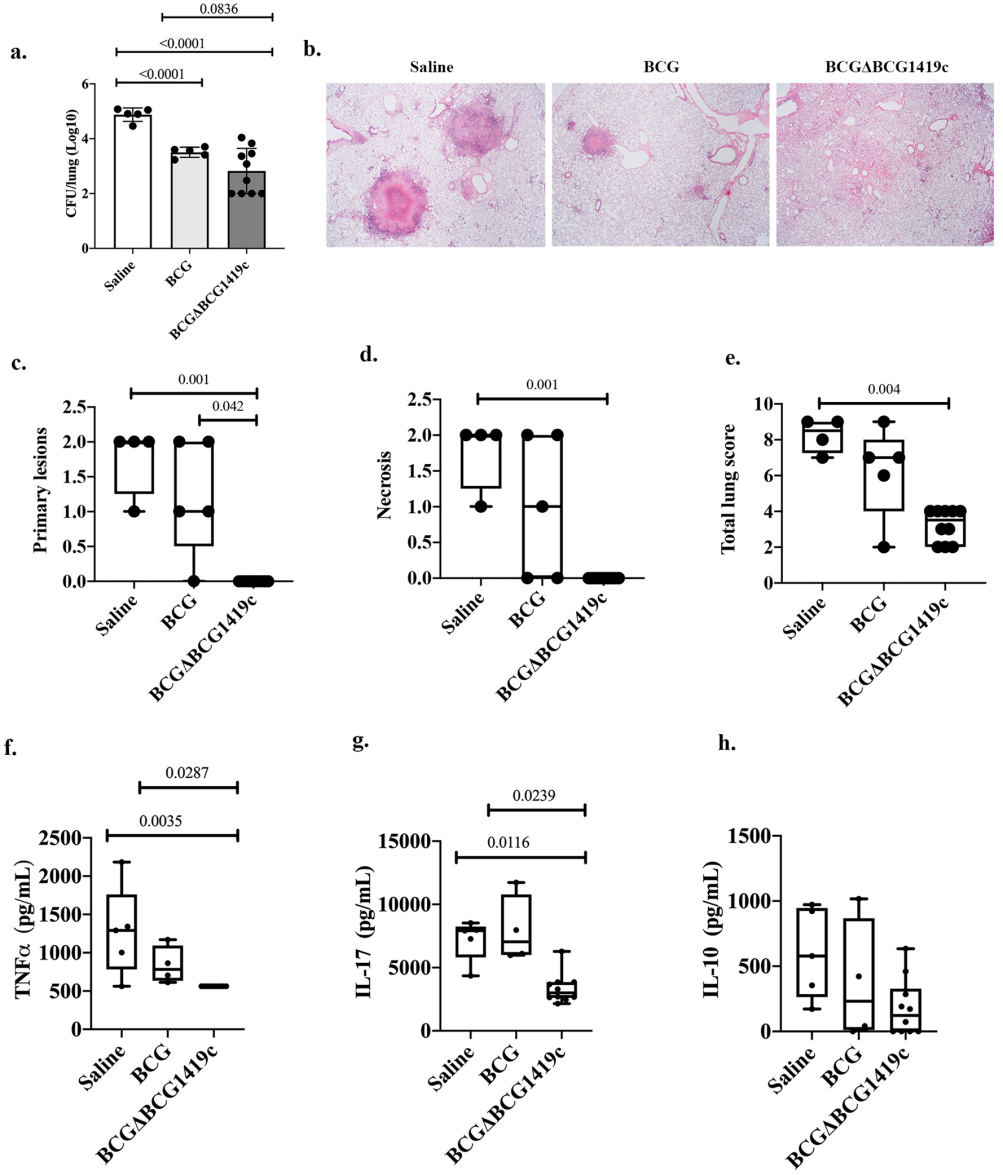
The BCGΔBCG1419c vaccine candidate is more effective than BCG in reducing pulmonary TB in guinea pigs. (**a**) Bacterial replication in lungs of guinea pigs (n = 5 in saline and BCG groups, n = 10 in the BCGΔBCG1419c group) at 40 days post-infection. Mean Log_10_ CFU with SD are shown, including individual data. Brown-Forsythe and Welch ANOVA test followed by Dunnett’s multiple comparison was used to compare *M. tuberculosis* burden and p values are shown. (**b**) Representative H&E images (×2) of animals that received saline, BCG, or BCGΔBCG149c. Lesions were evaluated using a scoring system to compare them (**c**). Primary lesions. (**d**) Necrosis. (**e**) Total lung score. Data (n = 4–10, depending on groups) is presented as a box and whisker plot showing all points. Categorized data were analyzed with a H Kruskal–Wallis test using SPSS version 25 (α = 0.05). In all cases, p value was adjusted for multiple comparisons with Bonferroni correction. Secreted cytokines in lungs of infected guinea pigs at 40 days post-infection, showing (**f**) TNF-α, (**g**) IL-17, and (**h**) IL-10. Data (n = 4–10, depending on groups) is presented as a box and whisker plot showing all points. A One-Way ANOVA followed by Tukey’s multiple comparison test or a Kruskal–Wallis followed by Dunn’s multiple comparison test were used depending on distribution of data, and significantly different p values are indicated.

To determine how effective were the BCG strains being evaluated in this work in controlling pulmonary TB, at 10 weeks post-vaccination with BCG or BCGΔBCG1419c, or mock vaccination with sterile saline solution, guinea pigs received a low dose (10–20 CFU) of *M. tuberculosis* H37Rv via aerosol. At 40 days post-infection, we found that both BCG- and BCGΔBCG1419c-vaccinated animals significantly reduced *M. tuberculosis* burden in lungs compared with those that received saline (1.3-log_10_ and 2-log_10_ respectively, p < 0.0001, Brown-Forsythe and Welch ANOVA test followed by Dunnett’s multiple comparison, Fig. 4a). The difference in control of *M. tuberculosis* replication in lungs between BCG and BCGΔBCG1419c showed a trend towards statistical significance when all groups were compared (Vaccinated and not vaccinated, Brown–Forsythe and Welch ANOVA test followed by Dunnett’s multiple comparison, p = 0.0836, Fig. 4a). When the comparison was solely between vaccinated groups, BCGΔBCG1419c reduced more than BCG the loads in lungs (p = 0.0308, unpaired, two-tailed Student’s t test with Welch’s correction).

A relevant feature of the first-generation, Hyg^R^ version of BCGΔBCG1419c is the reduction of lung pathology, compared to mice that received BCG^9,10^. To test whether this clinically relevant phenotype was conserved in the novel version of BCGΔBCG1419c, we compared its capacity to reduce lung pathology to that of BCG, and found that in several instances, BCGΔBCG1419c outperformed parental BCG. For instance, in animals receiving saline, we detected primary lesions with central necrosis surrounded by a rim of epithelioid macrophages and lymphocytes, as well as secondary lesions with a mixture of epithelioid macrophages and lymphocytes (Fig. 4b). When they received BCG, primary lesions with central necrosis were still observed, similar to those of unvaccinated animals, with secondary lesions of small size (Fig. 4b). When guinea pigs were vaccinated with BCGΔBCG1419c, we observed minimal alveolar and interstitial infiltration of macrophages and lymphocytes, but no granuloma formation (Fig. 4b). Moreover, when we applied a scoring system to quantitatively assess damage, we found that BCGΔBCG1419c significantly reduced primary lesions (p = 0.001 and p = 0.042 compared with saline and BCG-vaccinated animals, respectively, Fig. 4c), necrosis (p = 0.001 compared with saline group, Fig. 4d), and total lung score (p = 0.004 compared with saline group, Fig. 4e). Meanwhile, BCG did not significantly reduce necrosis nor total lung score.

To further characterize the effect of vaccination on pulmonary TB pathogenesis, we evaluated the production of TNF-α, IL-17, and IL-10 in lungs of vaccinated/non-vaccinated and infected guinea pigs. Here, we found that BCGΔBCG1419c led to a significant reduction of both TNFα (p = 0.0035 and p = 0.0287 compared with saline and BCG-vaccinated animals, respectively, Kruskal–Wallis followed by Dunn’s multiple comparison test, Fig. 4f) and IL-17 (p = 0.0116 and p = 0.0239 compared with saline and BCG-vaccinated animals, respectively, Kruskal–Wallis followed by Dunn’s multiple comparison test, Fig. 4g), while IL-10 remained without significant changes (Fig. 4h). Taken together, these results demonstrate that vaccination of guinea pigs with BCGΔBCG1419c led to improved control of pulmonary TB pathogenesis compared with BCG.

### BCGΔBCG1419c is more effective than BCG in reducing extrapulmonary TB pathogenesis

**Figure 5:**
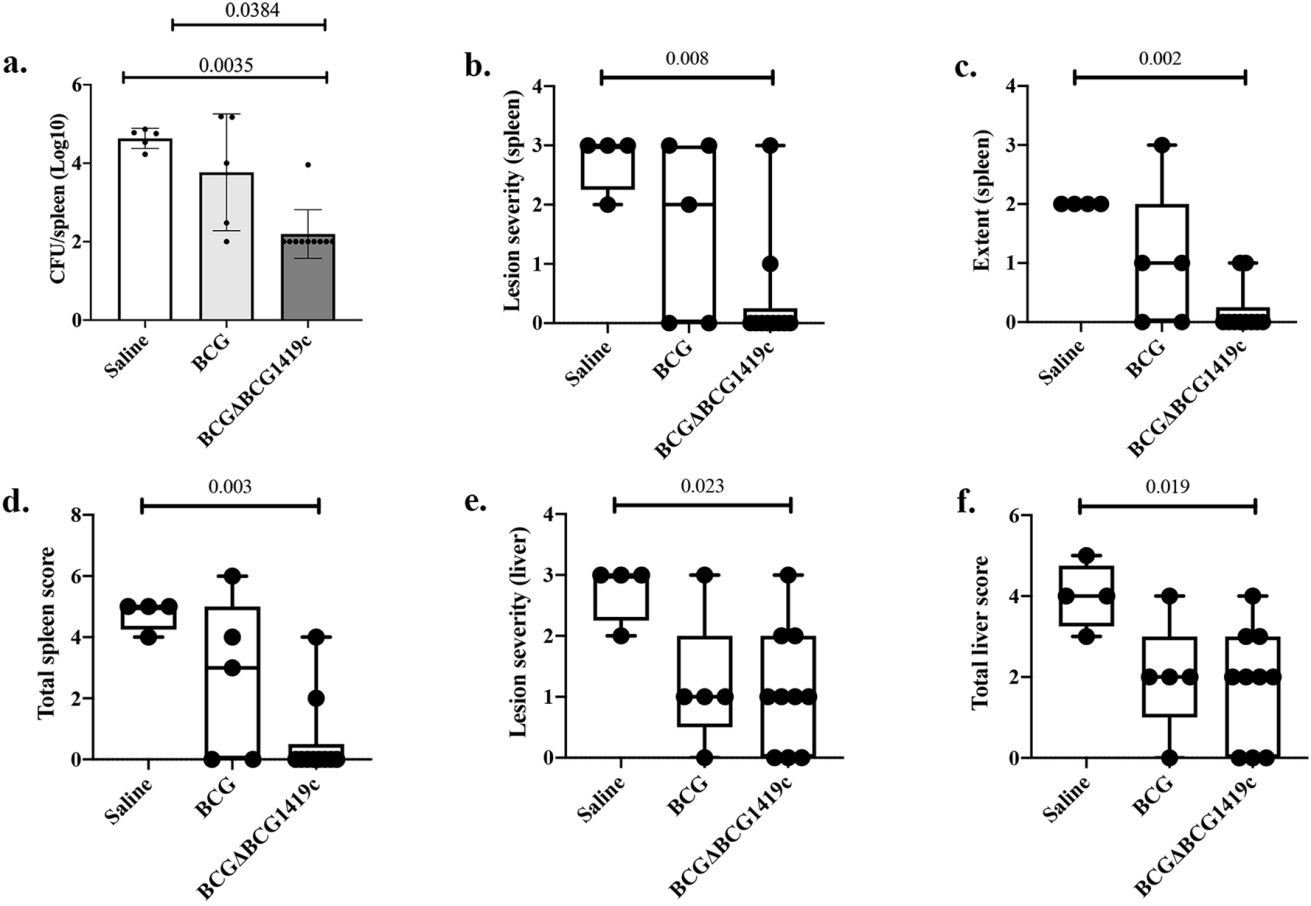
The BCGΔBCG1419c vaccine candidate is more effective than BCG in reducing extrapulmonary TB in guinea pigs. (**a**) Bacterial replication in spleens of guinea pigs (n = 5 in saline and BCG groups, n = 10 in the BCGΔBCG1419c group) at 40 days post-infection. Mean Log_10_ CFU with SD are shown, including individual data. A Kruskal–Wallis test followed by Dunn’s multiple comparison was used to compare *M. tuberculosis* burden and significant p values are shown. Lesions were evaluated using a scoring system to compare (**b**) Lesion severity in spleens. (**c**) Extent of lesions in spleens. (**d**) Total spleen score. (**e**) Lesion severity in livers. (**f**) Total liver score. Data (n = 4–10, depending on groups) are presented as a box and whisker plot showing all points. Categorized data were analyzed with a H Kruskal–Wallis test using SPSS version 25 (α = 0.05). In all cases, p value was adjusted for multiple comparisons with Bonferroni correction.

For yet not fully understood reasons, BCG is known to be more effective against childhood tuberculous meningitis and miliary tuberculosis than pulmonary TB^19^. Because of this, we evaluated the capacity of BCGΔBCG1419c and BCG to reduce *M. tuberculosis* CFU and pathology in spleens, and pathology in livers of aerosol-infected guinea pigs. We observed that in spleens, only BCGΔBCG1419c controlled replication of *M. tuberculosis,* when compared to either saline or BCG-vaccinated animals (p = 0.0035 and p = 0.0384, respectively, Kruskal–Wallis followed by Dunn´s multiple comparison test, Fig. 5a). Further to this, only BCGΔBCG1419c significantly reduced lesion severity (p = 0.008 compared with saline group, Fig. 5b), extent of damage (p = 0.002 compared with saline group, Fig. 5c), and total spleen score (p = 0.003 compared with saline group, Fig. 5d). Finally, in livers, only BCGΔBCG1419c significantly reduced lesion severity (p = 0.023 compared with saline group, Fig. 5e) and total liver score (p = 0.019 compared with saline group, Fig. 5f). As it occurred in lungs and spleens, BCG did not significantly reduce any of these pathological effects of TB in guinea pigs, therefore demonstrating that vaccination of guinea pigs with BCGΔBCG1419c led to improved control of extrapulmonary TB pathogenesis compared with BCG.

## Discussion

The two most-advanced live attenuated TB vaccine candidates, MTBVAC and VPM1002 have shown promising results in diverse preclinical models^20,21^. Both MTBVAC and VPM1002 are genetically modified, attenuated, antibiotic-less whole live mycobacteria, as it is our novel BCGΔBCG1419c vaccine candidate reported here. In this work, we observed a similar in vitro growth profile to its parental BCG. In addition, we recently reported that this novel version of BCGΔBCG1419c maintain changes in antigenic proteins compared with BCG^11^, therefore fostering our interest in its preclinical characterization. Among these changes in antigenic proteins, compared with wild type BCG, the BCGΔBCG1419c vaccine candidate increased secretion of GroEL1, DnaK and GroES^11^, which might contribute to an increased recognition by immune cells prompting a more efficient clearance of BCGΔBCG1419c in vivo (Fig. 2). On the other hand, the Hyg^R^ version of BCGΔBCG1419c lacked PDIM^8^, a common cause of attenuation in *M. tuberculosis* even during in vitro passages^22^, therefore providing another potential explanation for its attenuation, although we did not verify whether the antibiotic-less version of BCGΔBCG1419c also failed to produce PDIM.

In the guinea pig model, vaccination with 10^5^ CFU of MTBVAC showed similar efficacy to that of BCG Danish in reducing *M. tuberculosis* burden in lungs, as well as reducing lung and spleen pathology at 4 weeks post-infection^23^. Extending the time between vaccination and challenge from 112 to 210 days, led to an improvement in control of replication (1-log_10_ drop compared with BCG) but showed no difference in control of lung or spleen pathology^23^. Furthermore, MTBVAC showed similar efficacy to BCG for reduction of CFU in spleens, regardless of time post-vaccination evaluated, and the mean lung pathology score was similar to that afforded by BCG, regardless of time post-vaccination evaluated^23^. Based on this, depending on the time span between immunization and challenge, MTBVAC showed similar or improved control of either *M. tuberculosis* replication or infected-organ pathology, but not of both parameters in guinea pigs.

Here, we observed that guinea pigs vaccinated with the novel version of BCGΔBCG1419c (10^3^ CFU), improved control of both pulmonary and extrapulmonary TB more than its parental BCG Pasteur. Regarding control of pulmonary TB, we found that BCGΔBCG1419c-vaccinated guinea pigs reduced by approximately 0.7-log_10_ the mean CFU burden in lungs compared with BCG Pasteur at 40 days post-infection (Fig. 4a). In lungs, BCGΔBCG1419c-vaccinated guinea pigs had significantly reduced levels of TNFα and IL-17 compared with both BCG- and non-vaccinated animals (Fig. 4f,g). It is known that BCG vaccination allows guinea pigs to modulate TNF-α levels in conjunction with a reduction in bacillary loads in their tissues^24^. Regarding IL-17, genetic polymorphisms in this gene have been associated with TB susceptibility in humans^25^, but, on the other hand, Th17/IL-17 responses have been associated with TB pathogenesis and disease progression in other studies^26,27^. Based on our findings, we think that BCGΔBCG1419c-vaccinated guinea pigs had reduced TNFα and IL-17 in lungs because of the low *M. tuberculosis* burden, leading to reduced pathology in this group. Reduced IL-17 may possibly indicate a mechanism to dampen inflammation and neutrophil influx, which has been argued as the basis of the eventual lung necrosis^28^. In fact, guinea pigs with type 2 diabetes (T2D) showed increased lung pathology with increased IL-17 expression compared to non-diabetic controls^29^, further suggesting a link between increased levels of this cytokine and increased lung pathology.

Because the extent of extrapulmonary lesion necrosis was a better predictor of virulence than the number of viable bacilli in the tissue in guinea pigs infected with *M. tuberculosis*^30^, we think that it is of particular relevance that only BCGΔBCG1419c-vaccinated guinea pigs significantly reduced lung, spleen, and liver pathology scores compared with non-vaccinated controls, and primary lesions compared with BCG, although it also led to a significant 1.3-log_10_ reduction of the mean CFU in spleens (Figs. 4 and 5). Based on these results, in guinea pigs, BCGΔBCG1419c improved control of *M. tuberculosis* replication as well as reduced pulmonary and extrapulmonary TB pathology more than its parental BCG strain.

On the other hand, we observed higher anti-PPD IgG levels in BCGΔBCG1419c-vaccinated guinea pigs compared with those that received BCG, suggesting that the humoral response could play a role in protection afforded by the former strain. A number of mechanisms have been described for antibody-mediated immunity, including mycobacterial neutralization, antibody-dependent cellular phagocytosis (ADCP), complement activation, antibody-dependent cell-mediated cytotoxicity (ADCC), *M. tuberculosis*-antibody immune complex sensing by intracellular FcR tripartite motif-containing protein 21 (TRIM21), stimulation of cell-mediated immunity and modulation of the strength and nature of the inflammatory response during *M. tuberculosis*-infection (reviewed in Ref.^18^), therefore elucidating whether or not any of these possible mechanisms is involved in BCGΔBCG1419c-elicited protection remains a matter of future investigation.

 Taken together, based on a comparison of efficacy data reported for Hartley guinea pigs, we observe that BCGΔBCG1419c show similar or greater protection than that afforded by MTBVAC, although we acknowledge that this is based on a literature comparison only. Regarding safety of the novel BCGΔBCG1419c, here we demonstrated that it was more attenuated than its parental BCG strain in murine RAW 264.7 macrophages and in both athymic and immunocompetent mice, as well as in guinea pigs while inducing similar dermal reactivity in this latter species. Attenuation in macrophages of the antibiotic-less version of BCGΔBCG1419c agrees with that observed for the Hyg^R^ mutant^12^, while its safety in athymic mice was significantly lower than that of BCG. Of note, BCGΔBCG1419 Hyg^R^ was as attenuated as its parental BCG in athymic mice^9^, and we did not evaluate its safety in guinea pigs. As noted in Fig. 1a, even though some differences in gene deletion exists between the Hyg^R^ and antibiotic-less BCGΔBCG1419c versions, which could account for different attenuation, we think it is more likely that this is the consequence of using different parental BCG strains (ATCC 35734 here versus one kindly donated by James Triccas in Ref.^9^). This difference in BCG parental strains might contribute to explain why we did not detect an improvement in controlling pulmonary TB in guinea pigs for the BCGΔBCG1419 Hyg^R^ compared with wild type BCG, although the former strain did outperform BCG in reducing dissemination to spleen at 60 days post-infection^31^. It should be noted, though, that for technical reasons, when BCGΔBCG1419 Hyg^R^ was tested in guinea pigs, two doses of both BCG strains needed to be applied to be able to reach the planned 10^4^ viable CFU dose, therefore raising questions as to what extent did this contribute to the differences in control of pulmonary TB observed between Aceves-Sánchez et al.^31^ and this work.

We consider that it would be interesting to see whether increasing the time between vaccination with BCGΔBCG1419c and challenge, or vaccine dose, has any effect on its efficacy, as it occurred with MTBVAC^23^.

Another subject that might be worth investigating is whether intravenous vaccination increases the efficacy of BCGΔBCG1419c against TB, as it occurred in non-human primates vaccinated with BCG^32^. We think that a head-to-head comparison in the same experiment would shed light on the relative efficacy of BCGΔBCG1419c, VPM1002 and/or MTBVAC, allowing refinement of the current pipeline of TB vaccine candidates.

## Methods

### Mycobacterial strains

*Mycobacterium bovis* BCG Pasteur ATCC 35734 (hereafter referred to as BCG), or its isogenic derivative, second generation *M. bovis* BCGΔBCG1419c^11^ were cultured in Middlebrook 7H9 broth supplemented with 0.2% glycerol, 10% OADC, and 0.05% Tween 80, at 37 °C, 5% CO_2_, 100 rpm, up to an OD600nm ≈ 0.8, and used for safety studies in mice and guinea pigs, evaluation of antibody response in guinea pigs, RNA isolation as described^10^ and real time qPCR assays to determine relative expression of the *sigA* and *BCG1419c* genes to that of *rrs* as recently reported^33^. For evaluation of efficacy in guinea pigs, BCG and BCGΔBCG1419c were cultured in Proskauer and Beck (P&B) medium with 0.1% Tween 80 to mid-log phase. Aliquots were stored at − 80 °C and thawed before use. *M. tuberculosis* H37Rv (TMCC 102) was initially grown for three passages as a pellicle on P&B medium to produce seed stocks. Working stocks with a maximum of six passages were expanded from the seed stocks in P&B medium with 0.1% Tween 80. Working stocks were prepared at the mid-log phase, and aliquots were stored at − 80 °C and thawed before use.

### Macrophage infection and assessment of replication of the BCG strains 

This was performed essentially as described^12^. Briefly, 10,000 RAW 264.7 cells per well were grown in 96-well plates in 200 μL DMEM 2% FBS and antibiotic–antimycotic solution 1×. Once removed this media, cells were infected with 100 μL of BCG Pasteur or BCGΔBCG1419c, suspended in DMEM 2% FBS (MOI = 10). The infected macrophages were incubated at 35 °C with 5% CO_2_ for 6 h (considered as time zero post-infection). Then, the macrophages were washed three times with PBS solution, and 200 μL per well of fresh DMEM 2% FBS with antibiotic–antimycotic solution 1× were added to further incubate the cells. Samples were taken at 24 and 72 h post-infection. The assay was repeated with 3 replicates by infection/sample, in two independent experiments (n = 6 samples/BCG strain). To determine CFUs at each time point, we added 100 μL per well of 0.1% Triton X-100 and incubated at 37 °C in 5% CO_2_ for 15 min. The lysates were serially diluted into 0.05% PBS-Tween 20 solution, and 10 μL were inoculated onto 7H10 OADC agar plates, in duplicate. The plates were incubated at 37 °C in 5% CO_2_ for 4 weeks to perform the corresponding colonies enumeration.

### Safety studies in mice

Pathogen-free, 8–9 weeks old, female BALB/c, C57BL/6, and athymic (CAnN.Cg-*Foxn1*^nu^/Crl) nude mice were obtained from Bioterio Morelos (Mexico). Mice were maintained in vented cages with high-efficiency air-filters, with food and water ad libitum. Mice were randomly allocated to receive either BCG or BCGΔBCG1419c. BALB/c and C57BL/6 mice (n = 10/group) were immunized subcutaneously in the base of the tail with 2.5–2.7 × 10^7^ Colony Forming Units (CFU) of either BCG or BCGΔBCG1419c, suspended in 100 μL of sterile saline. The dose recommended for BCG in humans is 1–8 × 10^5^ CFU, then we used between 30-250 times the equivalent of a human dose. Weight was registered on a weekly basis up to 2 months post-vaccination, where CFU were determined in lungs and spleens, after euthanasia by cervical dislocation. For CFU enumeration, whole lungs or spleens from 5 mice per group were lysed and serial dilutions of the lysates were used for culture onto 7H9 OADC plates with 0.5% glycerol, incubated for 4 weeks at 37 °C, 5% CO_2_.

For safety studies in immunocompromised hosts, athymic mice (n = 10) received a single intravenous dose of 1 × 10^6^ CFU of either BCG or BCGΔBCG1419c, suspended in 100 μL of sterile saline. The primary endpoint of the experiment was defined as survival up to 180 days post-inoculation of animals vaccinated with BCG compared with those receiving BCGΔBCG1419c. At this time, surviving animals were humanely euthanized and bacterial load in lungs and spleen was quantified, as secondary endpoint. As a measure of health, weight was registered on a weekly basis. When any individual mouse reached a weight loss ≥ 20% of its maximum value, this was used as humane endpoint for euthanasia, by cervical dislocation. Whole lungs and spleens were lysed with Polytron (Kinematica, Luzern, Switzerland) in isotonic saline, and four dilutions of each homogenate were spread onto duplicate plates containing Middlebrook 7H10 agar (Difco Labs, Detroit MI, USA) enriched with 10% OADC. Plates were incubated for 4 weeks at 37 °C, 5% CO_2_. Weight registry and CFU enumeration were performed in a blind manner with respect to what BCG was applied to each animal.

### Excessive dermal reactivity in Guinea pigs

This assay was conducted as reported by Fitzpatrick et al.^34^. Briefly, for each of the BCG strains tested, a group of six pathogen-free, female outbred Hartley guinea pigs (200–250 g) was used (12 animals total), which were obtained from Bioterio Morelos (Mexico). Animals were maintained in vented cages with high-efficiency air-filters, with food and water ad libitum. Each guinea pig was injected intradermally with 10^4^, 10^5^, and 10^6^ CFU of BCG or BCGΔBCG1419c. A randomization plan was used to determine the injection sites for the doses (front, middle, or hind on either left or right flank of each guinea pig). The endpoint of the experiment was defined as the size of the lesions formed at the site of injection with BCGΔBCG1419c compared with that of BCG application, which were observed for 30 days and registered at regular intervals as indicated in “Results”. For each animal, the papule sizes induced by the BCG strains were compared at each dose applied. At the end of the experiment (30 days post-vaccination), animals were euthanized with sodium pentobarbital (150 mg/kg, intravenous, i.v.), The operator registering papule sizes was without prior knowledge of the BCG strain being applied at each site.

### Absence of virulent mycobacteria in Guinea pigs

This assay was conducted as reported by Fitzpatrick et al.^34^. Briefly, groups of six pathogen-free, female outbred Hartley guinea pigs (weight range 200–250 g) were used, which were obtained from Bioterio Morelos (Mexico). Animals were maintained in vented cages with high-efficiency air-filters, with food and water ad libitum. Each guinea pig was injected intramuscularly with 1.5 × 10^7^ CFU of either BCG or BCGΔBCG1419c (appromiately 100X the dose used in humans). Animals were weighed weekly and observed daily for 42 days for any TB symptoms. The primary endpoint of the experiment was defined as signs of TB symptoms, which will result in weight loss. As secondary endpoint, we determined lung pathology at 42 days post-vaccination in animals that received these BCG strains. At the end of this time, animals were euthanized with sodium pentobarbital (150 mg/kg, i.v.), and examined by necropsy. For pathological evaluation, both lungs were obtained from 4 guinea pigs and were perfused with 10% formalin in PBS pH 7.2. Once fixed, lungs were included in paraffin blocks and histological sections of 4 μM were obtained for both lungs and stained with Hematoxylin and Eosin (H&E). Histological and morphometric analysis was performed on these preparations by determining the percentage of pneumonic involvement using the Leica Application Suite v4.3 software. Pneumonic involvement is calculated as follows: the total area of the histological section is measured; this gives us a total area in square microns (μM^2^). Then, the area covered by the large blood vessels, bronchi and bronchioles is measured, these are considered spaces where there is no lung tissue (that is, the space of those vessels and bronchi), the result of this measurement is subtracted from the area of all the tissue previously measured, and this gives us a lung area that we consider as 100%.

Then, the area occupied by pneumonia is measured, defined as the cluster of proinflammatory cells (macrophages, neutrophils, lymphocytes, monocytes, epithelioid cells, and protein secretions) with undefined borders, which affect the lung structure, preventing adequate gas exchange. The percentage of pneumonia is then calculated with respect to the total area, per sample. Weight registry, histological and morphometric analysis were performed in a blind manner with respect to what BCG was applied to each animal.

### Recombinant protein expression and purification

*E. coli* BL21(DE3) harboring pMRLB.41 was cultured in 500 mL of LB /Amp (100 μg/mL) at 37 °C with shaking at 260 rpm until reaching an OD600 = 0.5, to add sterile IPTG (Sigma, USA) at a final concentration of 0.5 mM, followed by further incubation at 37 °C, 300 rpm for 4–6 h. Cells were pelleted by centrifugation at 4 °C, 10,000 rpm for 20 min, to store the pellet at − 20 °C until lysis. For bacterial lysis, 5 mL of binding buffer (20 mM Tris–HCl, 500 mM NaCl, 5 mM Imidazole, pH 7.9) were added per each gram of cell pellet, which was resuspended and sonicated with a probe (Sonics, USA) at level 5 for 15 s, with 40 s rest, for 15 cycles. After lysis, the material was centrifuged at 10,000 rpm for 20 min, recovering the supernatant and transferring it to a new 50 mL tube for a second round of centrifugation to remove debris and produce the soluble fraction, to which Protease inhibitor cocktail (Sigma, EU) was added at a final concentration of 0.1× and store at − 20 °C until use.

For purification of His-tagged, recombinant Ag85A, 1 mL of HisPur ™ Ni-NTA Resin (Thermo Fisher Scientific, USA, Catalog 88221) was placed in a column (BioRad, USA), equilibrated with 10 column volumes (CV) of binding buffer and the resin was incubated with the soluble fraction at 4 °C with gentle shaking overnight. The resin with bound protein was washed with 6 CV of washing buffer (20 mM Tris–HCl, 500 mM NaCl, 60 mM Imidazole, pH 7.9), followed by 10 CV of 10 mM Tris–HCl. Next, 10 CV of 0.5% of ASB-14 in 10 mM Tris–HCl were used to remove endotoxins, followed by 10 CV of 10 mM Tris–HCl to remove residual ASB-14. To recover the purified recombinant proteins, five steps of elution were performed, each one where 1 mL of elution buffer (10 mM Tris–HCl, 1 M Imidazole, pH 8) was incubated with the resin for 5 min, to elute and recover in 1.5 mL tubes. Presence and apparent purity of the recombinant proteins within the eluted fractions were confirmed by SDS-PAGE in 15% gels stained with brilliant blue G (Sigma, USA). Fractions presenting the highest abundance and purity of the recombinant protein were combined and dialyzed using a membrane (SnakeSkin™ Dialysis Tubing, 10 K MWCO, 22 mm) (Thermo Scientific, EU), immersed in 1 L of 1X PBS (in MilliQ water) and incubated overnight at 4 °C with gentle shaking, with two buffer exchanges every 2 h. Aliquots of dialyzed proteins were quantified in triplicate using the micro BCA kit (BioVision, EU, Catalog K813), with samples read at 595 nm in a 680 XR microplate reader (BioRad, EU), by comparison with a standard curve of BSA (Sigma, USA, from 3 to 1500 μg/mL). LPS content was verified with the Pierce Chromogenic Endotoxin Quantitation Kit (Thermo Fisher, Cat A39553) according to the manufacturer’s instructions, and found to be 0.7 EU for Ag85A in 50 ng of its His-tagged recombinant forms. 100 μL aliquots of purified and dialyzed proteins were stored at − 20 °C until further use, to avoid repeated freeze–thaw cycles.

### Evaluation of antibody response in vaccinated guinea pigs

Three pathogen-free, female outbred Hartley guinea pigs (weight range 200–250 g) were used per group, which were obtained from Bioterio Morelos (Mexico). Animals were maintained in vented cages with high-efficiency air-filters, with food and water ad libitum. Each guinea pig was injected intradermally with 10^3^ CFU of either BCG or BCGΔBCG1419c. After 70 days, blood was drawn to obtain sera. The primary endpoint of the experiment was defined as anti-PPD IgG and anti-Ag85A levels in guinea pigs vaccinated with BCG compared with those receiving BCGΔBCG1419c. For this, IgG antibodies were detected by Enzyme-Linked ImmunoSorbent Assay (ELISA). For this, 50 ng of either bovine PPD (PRONABIVE, obtained from *Mycobacterium bovis* AN5) or His-tagged Ag85A, were added to each well of Costar 96 well plates (Corning, USA, catalog 3590) and incubated at 37 °C for 1 h. Then, 150 µL of 3% BSA in PBS 1× were placed for incubation at 37ºC for 1 h. This blocking solution was discarded and then 50 µL of guinea pig sera (1:5 in PBS 1×) were added and incubated at 37 °C for 1 h. Each well was washed three times with 200 µL of PBST (PBS 1×, 0.05% Tween 20), to add 50 µL of an HRP-conjugated, rabbit anti-guinea pig IgG secondary antibody (Invitrogen, USA, catalog 61-4620) diluted 1:2000 in 1% BSA-PBS 1×, to further incubate at 37 °C for 1 h. After washing three times, 50 µL of 1-Step TM Ultra TMB-ELISA (Thermo Scientific, USA, catalog 34028) were added. Five minutes after adding the substrate, 50 µL of sulfuric acid 0.5 M were added to stop the reaction, and the optical density (OD) at 450 nm was determined using a microplate spectrophotometer (xMark Biorad, USA). ELISA analysis was performed in a blind manner with respect to what BCG was applied to each animal. Values reported are after subtracting the OD450 of control wells with no sera added.

### Protection in the Guinea pig aerosol challenge model

Outbred Hartley guinea pigs were maintained in an ABSL-3 facility at Colorado State University, with sterile chow and water ad libitum. Five or ten animals per group, depending on the group (approximately 450–500 g, Charles River Laboratory) were injected subcutaneously with a microchip for identification and assessment of body temperature, which was measured to track the clinical progression of the disease. The microchip implant (IPT- 300 Bio Medic Data Systems [BMDS], Inc., Seaford, DE) allowed daily measurement of body temperature and also carried information about experiment number and animal number. The body temperature of individual guinea pigs was assessed each weekday at approximately the same time using a DAS-6006/7 scanner transponder (BMDS). Guinea pigs were placed into groups of five or ten, with control group receiving sterile pyrogen-free saline, while experimental groups received 10^3^ CFU via the intradermal route of BCG or BCGΔBCG1419c.). At this time post-vaccination, guinea pigs were infected with a low dose aerosol (10–20 CFU) of virulent *M. tuberculosis* H37Rv using the Madison Aerosol Exposure Chamber (University of Wisconsin, Madison, WI). The body weight of each guinea pig was assessed weekly. The endpoint of the experiment was defined as protection against *M. tuberculosis* challenge conferred in vaccinated versus non-vaccinated animals. For this, at day 40 post-infection and upon necropsy, CFU, pathology (lung, spleen, and liver), and cytokine ELISA were determined.

### CFU, pathology assessment in infected organs, and cytokine ELISA in lungs

The right caudal lobe of the lung and a piece of the spleen and liver from the guinea pig was utilized to analyze pathological lesions. The excised lung lobe was inflated with formalin and placed in total into formalin. For processing, the lung lobe, spleen, and liver were embedded in paraffin and sections cut and stained with H & E. A pathologist examined the sections, without prior knowledge of the groups, and provided a score based on the extent of lung involvement, fibrosis, and lesion type. The right cranial lobe of the lung and half of the spleen were sampled to assess CFU numbers. Bacterial load was determined by plating serial dilutions of organ homogenates onto nutrient 7H11 agar supplemented with OADC. Colonies were enumerated after 3 weeks of incubation at 37 °C and are expressed as log_10_ transformed data. For lung, spleen, and liver pathology, we used a score based on the extent of organ involvement, inflammation, granuloma formation, necrosis, mineralization, and fibrosis and lesion type as previously described in detail^35^. Lung homogenates that were used for determining CFU were also used to assess IFN-γ, TNF-α, IL-17 and IL-10. IL-17 and TNF-α kits were purchased form Kingfisher Biotech (Saint Paul, MN) and the IL-10 kit was purchased from Reddot (Kelowna, BC, Canada).

### Ethical statement

For safety experiments in mice and guinea pigs, the local animal ethics committee approved all experiments, which were performed following Mexican guidelines regarding ethical and safe handling of experimental animals such as: NOM-07- SEMARNAT-SSA1-2002, NOM-033-ZOO-1995, and NOM-062- ZOO-1999. Experiments with nu/nu mice and Hartley guinea pigs were conducted under permit 2020-008A. Experiments with BALB/c and C57BL/6 mice were conducted under permit 2020-001C. For experiments with guinea pigs at Colorado State University, the Institutional Animal Care and Use Committee (IACUC) approved all experimental procedures under permit IACUC: #1206.

### Adherence to ARRIVE guidelines

All protocols involving animals were performed according to the ARRIVE guidelines 2.0 (https://arriveguidelines.org/arrive-guidelines), where the essential 10 and recommended set of details were indicated per specific experimental approach.

### Statistical analyses

Unless indicated otherwise, data are presented as means with standard deviations, median and ranges, or median with standard deviation. Distribution of data was determined with the Shapiro–Wilk test. Comparison among groups were performed in Prism v.8, according to their distribution (normal or non-normal) and number of groups being compared. For comparison of CFU evaluated in RAW macrophages, in vitro replication, and safety experiments performed in mice and guinea pigs, an unpaired, two-tailed Student’s t test was used to compare data from 2 groups, with Welch’s correction when standard deviation from the groups was different. A Mann–Whitney test was used to compare 2 groups with a non-normal distribution, as it was for the real time qPCR comparison between BCG and BCGΔBCG1419c. For comparison of *M. tuberculosis* CFU obtained in vaccinated versus non-vaccinated guinea pigs, CFU were log_10_ transformed and compared with a Brown-Forsythe and Welch ANOVA test followed by Dunnett’s multiple comparison test, or a Kruskal–Wallis test. To compare weight gain/loss and papule sizes, in animals receiving BCG or BCGΔBCG1419c, as well as for OD600nm comparison, a multiple t tests corrected for multiple comparison with Holm–Sidak method at α = 0.05 was used. For comparison of survival curves, the Log-rank (Mantel-Cox) test was used. Group comparisons where p < 0.05 were considered different. Categorized data (histological analyses) were analyzed with a H Kruskal–Wallis test using SPSS version 25 (α = 0.05). In all cases, p value was adjusted for multiple comparisons with Bonferroni correction.

## Data Availability

The datasets generated and analyzed in this study are available from the corresponding author upon reasonable request.
